# Nursing Support for Constipation in Palliative Care Units in Japan: A Multisite Cross-Sectional Study

**DOI:** 10.1089/pmr.2024.0087

**Published:** 2025-01-08

**Authors:** Kimiko Nakano, Yusuke Kanno, Kohei Kajiwara, Masamitsu Kobayashi, Miharu Morikawa, Yoshinobu Matsuda, Jun Kako

**Affiliations:** ^1^ Nursing Department, Mie University Hospital, Tsu, Japan.; ^2^ Graduate School of Health Care Sciences, Institute of Science Tokyo, Tokyo, Japan.; ^3^ Faculty of Nursing, Japanese Red Cross Kyushu International College of Nursing, Munakata, Japan.; ^4^ Graduate School of Nursing Science, St. Luke’s International University, Tokyo, Japan.; ^5^ Graduate School of Medicine, Kyoto University, Kyoto, Japan.; ^6^ Department of Psychosomatic Internal Medicine, NHO Kinki Chuo Chest Medical Centre, Sakai, Osaka, Japan.; ^7^ Department of Nursing, Graduate School of Medicine, Mie University, Tsu, Japan.

**Keywords:** cancer, constipation, nursing, support

## Abstract

**Purpose:**

This study investigated the frequency with which nursing support for constipation is provided for patients with cancer during the prognostic months and weeks.

**Methods:**

An online cross-sectional survey was conducted anonymously among registered nurses in palliative care units across Japan. The frequencies of providing six types of nursing support (abdominal massage with essential oils, abdominal acupressure, auricular acupressure, self-management education, abdominal massage, and warm compresses) were surveyed.

**Results:**

Data were obtained from 539 nurses (response rate: 22.3%) from 162 facilities. The most frequently provided support was warm compression; the least frequently provided supports were auricular acupressure, abdominal massage with essential oils, and abdominal acupressure. In the prognostic weeks only, self-management education followed these support types.

**Conclusion:**

The investigation found that the six types of support were rarely implemented for relieving constipation in patients with cancer. Future research should investigate the factors hindering the provision of these supports.

## Introduction

Constipation is an uncomfortable symptom experienced by patients with cancer, which reduces their quality of life.^1,2^ Constipation is managed by pharmacological and nonpharmacological therapies. However, few reports on effective nonpharmacological therapies for constipation exist.^3–7^ In a scoping review of nursing support types to relieve constipation in patients with cancer, aroma-based massage to the abdomen, auricular acupressure, abdominal acupressure, and self-management education were identified.^8^ Moreover, abdominal massage and acupressure have recently been shown to be effective in the management of opioid-induced constipation (OIC).^9,10^ In Japan, warm compresses are empirically used to relieve constipation and increase intestinal peristalsis, promote defecation and evacuation, and improve constipation.^11,12^

Nurses play an important role in providing nonpharmacological therapy. However, the nursing support that is provided based on prognosis is unclear. This study surveyed how frequently the nursing support types identified in previous studies are provided in palliative care units (PCUs) in Japan. By investigating the extent to which nursing support for constipation is provided based on prognosis, the gap between evidence and clinical practice can be identified. Moreover, it would provide useful information to nurses providing palliative care and contribute to future nursing support.

### Purpose

This study investigated the frequency with which nursing support for constipation is provided by registered nurses during the prognostic months and weeks in PCUs across Japan.

## Methods

### Study design

A multisite online cross-sectional survey was conducted anonymously among registered nurses in PCUs across Japan.

### Participant recruitment

First, a document explaining the research was sent to the administrators of all facilities with PCUs in Japan, and their participation was requested. After obtaining consent, a document describing the research was mailed to the nurses in the facilities, and individual access to an online questionnaire was granted. Only nurses who agreed to participate in the study completed the online questionnaire. Data were collected between October 2023 and March 2024 from the nurses who provided direct care in PCUs. The nurses completed the survey anonymously using LimeSurvey Cloud (LimeSurvey GmbH, cf. https://www.limesurvey.org/ja).

### Measurements

The survey was conducted based on the scoping review that aimed to extract evidence on nursing support to provide constipation relief to terminally ill patients with cancer.^6^ The identified support types were abdominal massage with essential oils, abdominal acupressure, auricular acupressure, and self-management education.^6^ In addition, a preliminary survey of nine nurses with PCU experience identified nursing support for abdominal massage and warm compresses. The questions described six types of nursing support and asked the same questions based on prognostic months and weeks. For example: “1. Based on your experience in providing abdominal massage for constipation to promote bowel movement, how much would you provide it in the following conditions: 1–1. The patient’s “prognostic months” situation? 1–2. The patient’s “prognostic weeks” situation?” All questions were scored on a Likert scale with the following options: “1. Never,” “2. Seldom,” “3. Sometimes,” “4. Frequently,” and “5. Always.”

In addition, demographic data, such as sex, age, educational background, qualifications, years of nursing experience, and years of PCU experience, were collected.

### Statistical analysis

Descriptive statistics were used to analyze demographic information and nursing support frequencies. EZR, a component of the R software (Saitama Medical Center, Jichi Medical University, Saitama, Japan), was used for statistical analysis.^13^

### Ethical considerations

This study was approved by the relevant clinical research ethics review committee (blinded for review). All the participants provided informed consent by responding affirmatively to a consent form. Reporting followed the Strengthening the Reporting of Observational Studies in Epidemiology guidelines.^14^

## Results

A request was sent to all 389 palliative care wards in Japan, of which 162 participated. A total of 2448 nurses were requested to complete the survey, with 539 responding (valid response rate: 22.3%). The participants’ demographics are shown in Table 1. Most were female (94.8%), with a mean age of 42.3 years (standard deviation [SD]: 9.8 years), and attended a vocational nursing school (including a junior college) (*n* = 456; 84.6%). The mean nursing experience was 18.6 years (SD: 9.6 years) and that in PCUs was 4.8 years (SD: 4.3 years). Most participants with specialty certifications were registered palliative care nurses (*n* = 29; 5.3%).

**Table 1.** Participants’ Demographics (*n* = 539)

	N	%
Sex		
Female	511	94.8
Male	26	4.8
Prefer not to answer	2	0.4
Educational background		
Nursing vocational school (including junior college)	456	84.6
Nursing university	64	11.9
Master’s program (pre-doctoral program)	10	1.9
Doctoral program (post-doctoral program)	1	0.2
Qualifications		
Certified nurse of palliative care	29	5.3
Certified nurse of cancer pain management nursing	4	0.7
Certified nurse specialist of cancer nursing	3	0.6
Qualifications (other)		
End-of-life care specialist	8	1.5
Certified public psychologist	2	0.4
Other	6	1.1
	Mean	SD
Age	42.3	9.8
Years of nursing experience	18.6	9.6
Years of experience in palliative care unit	4.8	4.3

SD, standard deviation.

Nursing support for constipation in patients with cancer, based on the prognosis in months and weeks, is shown in Table 2. Auricular acupressure was the least frequently implemented of the six types of support. The sum of the percentages of each nursing support based on prognosis and differences from month to week are shown in Table 3. The highest percentage of registered nurses who reported implementing interventions “frequently or more” provided warm compression; the lowest percentage implementing interventions “never or less” provided auricular acupressure, abdominal massage with essential oils, and abdominal acupressure. In addition, in the prognostic weeks only, self-management education followed these support types.

**Table 2.** Nursing Support for Constipation in Patients with Cancer with the Prognosis of Months and Weeks (*n* = 539)

	Months		Weeks	
Nursing support	n	%	n	%
Warm compress				
Always	81	15.0	78	14.5
Frequently	156	28.9	146	27.1
Sometimes	220	40.8	214	39.7
Seldom	49	9.1	64	11.9
Never	33	6.1	37	6.9
Abdominal massage^a^				
Always	56	10.4	46	8.5
Frequently	128	23.8	124	23.0
Sometimes	271	50.4	268	49.7
Seldom	62	11.5	81	15.0
Never	21	3.9	20	3.7
Self-management education				
Always	21	3.9	16	3.0
Frequently	86	16.0	43	8.0
Sometimes	261	48.4	174	32.3
Seldom	99	18.4	177	32.8
Never	72	13.4	129	23.9
Abdominal acupressure				
Always	6	1.1	4	0.7
Frequently	18	3.3	22	4.1
Sometimes	82	15.2	69	12.8
Seldom	95	17.6	102	18.9
Never	338	62.7	342	63.5
Abdominal massage with essential oils				
Always	10	1.9	10	1.9
Frequently	11	2.0	13	2.4
Sometimes	49	9.1	47	8.7
Seldom	112	20.8	109	20.2
Never	357	66.2	360	66.8
Auricular acupressure				
Always	4	0.7	5	0.9
Frequently	6	1.1	3	0.6
Sometimes	14	2.6	15	2.8
Seldom	31	5.8	28	5.2
Never	484	89.8	488	90.5

^a^ *n* = 538 due to one missing response.

**Table 3.** Sum of Percentages of Each Type of Nursing Support by Prognosis and Differences from Months to Weeks

Nursing support	Months	Weeks	Difference from months (%)
	%	%	
Warm compress			
Always Frequently	43.9	41.6	2.3
Sometimes	40.8	39.7	1.1
Never Seldom	15.2	18.8	−3.6
Abdominal massage			
Always Frequently	34.2	31.5	2.7
Sometimes	50.4	49.7	0.7
Never Seldom	15.4	18.7	−3.3
Self-management education			
Always Frequently	19.9	11	8.9
Sometimes	48.4	32.3	16.1
Never Seldom	31.8	56.7	−24.9
Abdominal acupressure			
Always Frequently	4.4	4.8	−0.4
Sometimes	15.2	12.8	2.4
Never Seldom	80.3	82.4	−2.1
Abdominal massage with essential oils			
Always Frequently	3.9	4.3	−0.4
Sometimes	9.1	8.7	0.4
Never Seldom	87	87	0
Auricular acupressure			
Always Frequently	1.8	1.5	0.3
Sometimes	2.6	2.8	−0.2
Never Seldom	95.6	95.7	−0.1

We calculated the sum of percentages of “Always” and “Frequently” and “Never” and “Seldom” for each type of nursing support, as well as the differences between months and weeks.

## Discussion

This study investigated nursing support for relieving constipation in patients with cancer in Japanese PCUs, with a focus on prognosis. We explored the differences in the frequency with which each type of nursing support is implemented in practice. The trends in nursing support were considerably similar for patients with a prognosis of weeks and months.

Auricular acupressure, abdominal massage with essential oils, and abdominal acupressure were rarely provided in both months and weeks of prognosis. The effectiveness of these nursing support types has been reported in the existing literature.^6^ Applicability of auricular acupressure was rated differently in an expert Delphi survey conducted prior to this study regarding application to patients with a prognosis of weeks.^15^ This may have been because of low access to knowledge and information and required time for support to become effective. The results suggest that access to such techniques is limited in Japan.

Warm compression was the most frequently provided support; however, it was provided only by ∼40% of all nurses, and the frequency of nursing practice was not high. Warm compression improves constipation and quality of life in women.^16^ It is an empirically common form of support in Japan but may not be used in other countries. Similarly, abdominal massage is an effective approach to relieve constipation in patients with OIC and increase bowel movements.^9^ These nursing support types are not expensive, are easy to provide, and can be offered in addition to pharmacotherapy.

Self-management education was sometimes provided in the prognosis months, but rarely in the prognosis weeks. In a previous study, self-management education was provided to patients undergoing chemotherapy for breast cancer who maintained their activities of daily living.^17^ The program consisted of abdominal massage, abdominal muscle stretching, and education on proper defecation positions. Abdominal muscle stretching was difficult to implement in prognostic weeks. Education regarding proper defecation position is also recommended by the Multinational Association of Supportive Care in Cancer and is useful for patients with a prognosis of weeks.^4^ Therefore, depending on the patient’s medical condition and status, it is necessary to promote self-management education to relieve constipation.

Effective nursing support for constipation is gradually being identified; however, knowledge of care providers and support systems in facilities is necessary to provide effective nursing support in clinical settings. In the future, it will be important to investigate the factors that make it difficult to provide nursing support to relieve constipation.

### Limitations

This study had several limitations. First, this survey was conducted online, and the response rate was low, possibly introducing a bias toward nursing practices of respondents who were enthusiastic about implementing the interventions. Second, nursing support such as hot compresses may be influenced by cultural backgrounds and is rarely implemented outside Japan. Although there are few studies on nursing support that are effective in relieving constipation, there may be other types of nursing support that are empirically implemented globally.

## Conclusion

This study investigated nursing support for relieving constipation in patients with cancer in Japanese PCUs, with a focus on prognosis. The study found that six types of support for relieving constipation were rarely implemented in palliative care wards in Japan. Future research is needed to investigate the factors hindering these nursing support types for constipation relief.

## Data Availability

The data were registered in the Individual Case Data Repository of the UMIN (University Hospital Medical Information Network), Japan.

## Abbreviations Used

OIC: Opioid-Induced Constipation
PCUs: Palliative Care Units
